# Femoral Nerve Palsy Following Delayed Reduction of a Dislocated Hip in a 44- Year-old Man

**DOI:** 10.5812/ircmj.12579

**Published:** 2014-02-05

**Authors:** Hassan Rahimi Shorin, Mohammad Azizbeig Mohajer, Ali Parsa, Amin Azhari, Maryam Assadian

**Affiliations:** 1 Department of Orthopaedic Surgery, Mashhad University of Medical Sciences, Mashhad, IR Iran; 2 Department of Orthopaedic Surgery, Graz University, Graz, Austria; 3 Department of Orthopaedic Surgery, Zahedan University of Medical Sciences, Zahedan, IR Iran; 4 Department of Medical physics and Rehabilitation, Mashhad University of Medical Sciences, Mashhad, IR Iran

**Keywords:** Dislocation, Hip, Femoral Neuropathy

## Abstract

**Introduction:**

Incidence of nerve injury in traumatic hip dislocations is up to 10 %. Sciatic nerve is the most common injured nerve in this setting. In the medical literature, there are few documented cases of femoral nerve injury following hip dislocations.

**Case Report:**

We report a 44-year-old man with right femoral nerve palsy following delayed reduction of an anterior dislocation of hip.

**Conclusion:**

Two months after closed reduction, complete clinical recovery of right femoral nerve was achieved and the patient was able to resume his job.

## 1. Introduction

Hip dislocation is either congenital or traumatic. Congenital dislocation of the hip is due to acetabular dysplasia as a result of immature acetabulum in infants, proximal femoral deformities, or dysplasia of the femoral head or acetabulum. On the other hand, traumatic dislocation often occurs during high-speed or high-energy accidents such as road-traffic accidents. A traumatic hip dislocation alone or with accompanying hip fracture is an orthopedic emergency, and it is advocated that an early reduction might reduce complications (1, 2). According to the previous studies, incidence of nerve injury in traumatic hip dislocations is up to 10 % and sciatic nerve is the most common injured nerve. In the medical literature, there are few documented cases of femoral nerve injury following hip dislocations. We report a case of femoral nerve palsy secondary to hip dislocation.

## 2. Case Report

**Figure 1.**
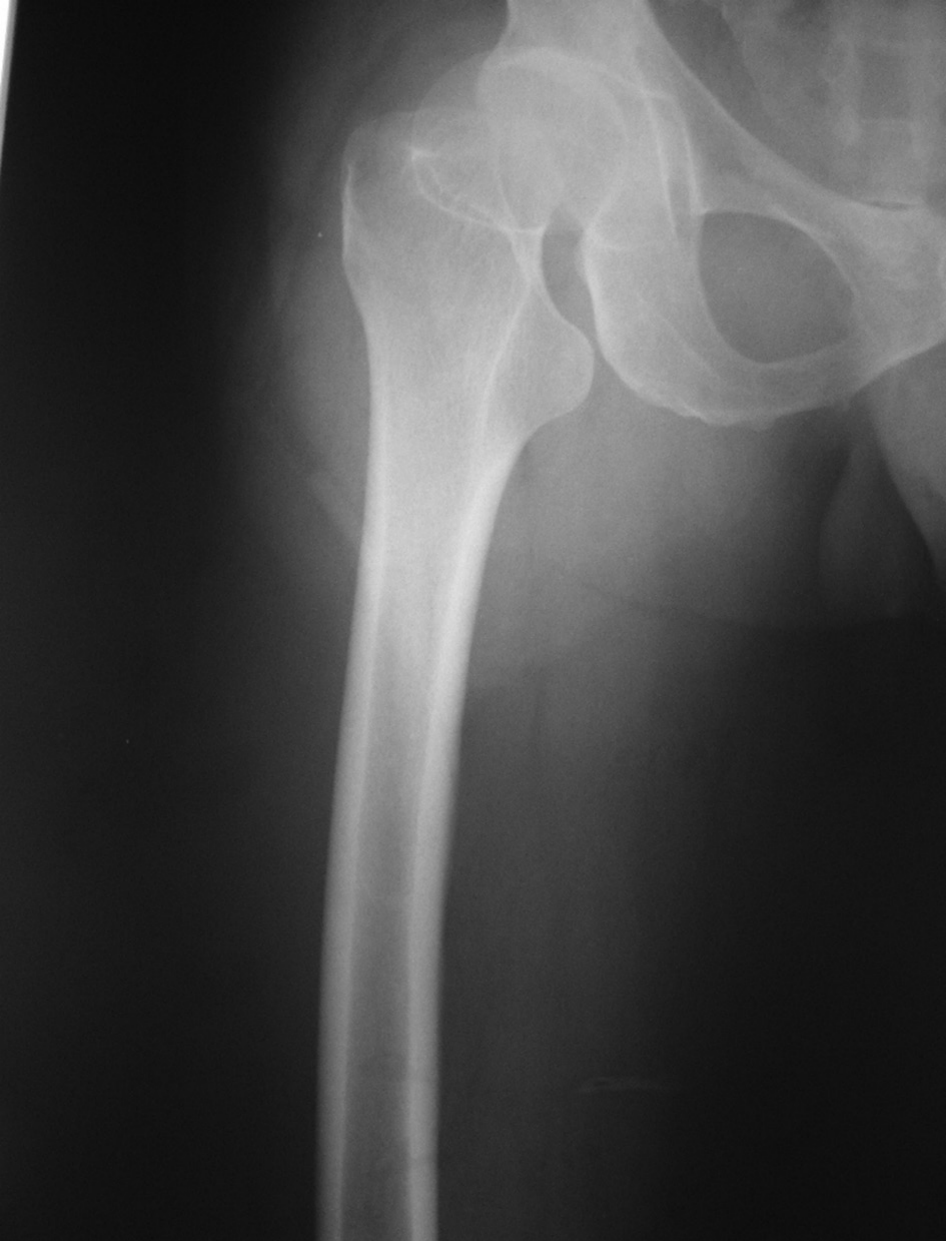
Anterior-Posterior X-ray Images of the Right Hip

**Figure 2.**
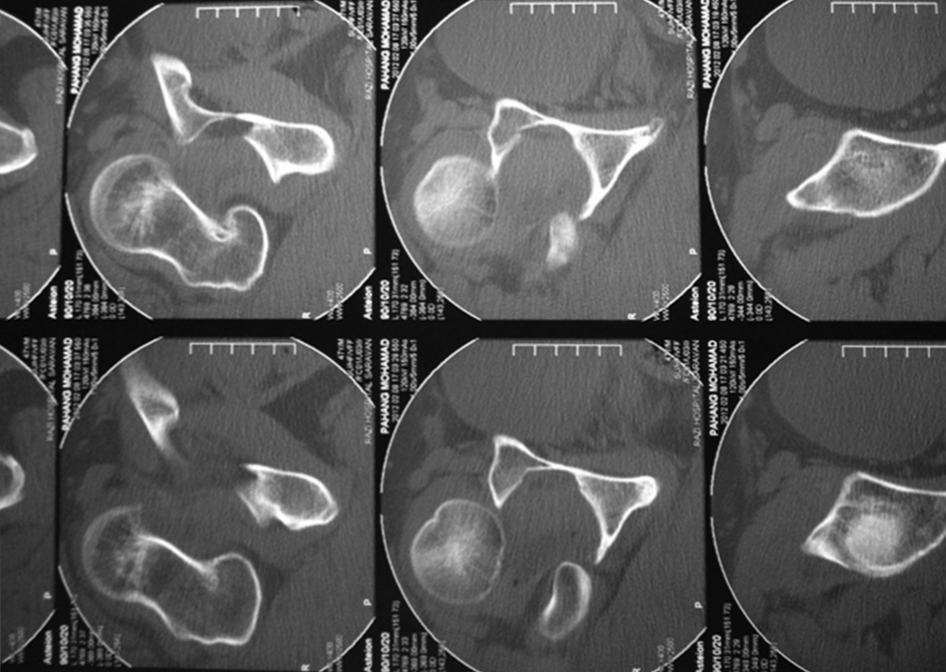
Computed Tomography of the Right Hip

In April 2012 a 44-year-old man pedestrian was referred to our hospital (Khatam-al-anbia, Zahedan, Iran) 18 hours after a motor-vehicle accident. First, He was transferred to the nearest hospital, but his dislocation was missed on initial X-ray images (Figure 1). Few hours later, a CT scan was performed owing to his increasing pain that revealed an anterior dislocation of his right hip joint (Figure 2). Consequently, he was referred to our center. In Emergency room, his right lower extremity was in external rotation position. He explained an anesthesia in medial side of right leg and ankle. Active extension of the right knee was impossible and the muscle force could not be assessed due to extreme pain. Motor and sensory functions of the sciatic nerve were normal. The right hip was reduced under general anesthesia in the theater and was followed by skin traction. Post-reduction radiographic images demonstrated an appropriate anatomical position of the right reduced hip without any fracture.

After reduction, marked wasting of knee extensor muscles and decreased sensation in the femoral nerve distribution were noted on examination. Electrophysiological studies shown a neurapraxia and the patient was informed that the muscles force would be restored and the numbness would be resolved. On discharge, skin traction for two weeks, gentle range of motion and knee extensors strengthening exercise, and analgesic were prescribed. Three weeks later, there has been a steady improvement in the patient's symptoms. He could extend the right knee against gravity and the sensation was regained in the medial of the right leg. Therefore, gait training was initiated and physical therapy was continued. At two months follow-up, complete clinical recovery of right femoral nerve was achieved and he was able to resume his job. Findings of an electromyography and nerve conduction study were normal at three months after injury. Recent eight months review has not shown any neurological deficit and he had full range of motion in both right knee and hip joints.

## 3. Discussion

Traumatic dislocation of the hip is an absolute orthopedic emergency. Prompt stable reduction is the key of successful management (3). A delay in detection and reduction leads to irreversible complications (1). While the presentation of sciatic nerve palsy following hip dislocation is well documented, there are few documented cases of femoral nerve palsy following hip dislocation. Other causes of femoral nerve paralysis include pelvic surgeries, retroperitoneal hemorrhage, displaced acetabular fractures (4), iliacus muscle hematoma (5), compartment syndrome (6), open wounds (7), treatment of the hip dysplasia with pavlik harness(8), congenital hip instability (9), and injury during total hip surgery (10, 11). The femoral nerve lies medial to the psoas muscle in the same sheath and can be injured with anterior dislocation of hip. In anterior dislocations, the psoas acts as the fulcrum of the hip and the capsule is disrupted both anteriorly and inferiorly. Although rare, in extremely high-energy injuries the femoral neurovascular bundle can be injured or an open dislocation can occur (9, 12). Up to December 2012, there were a few published articles concerning femoral nerve palsy secondary to hip luxation. Stein et al. reported hip dislocation of a ballet dancer during a non-contact activity with a post-injury femoral nerve neuropraxia that resolved within six weeks (13). Another patient with a posterior hip dislocation with femoral nerve palsy was reported by Frew et al. (14). In a large series consisted of 726 patients with displaced acetabular fractures, Konrad et al. reported only two cases of posttraumatic femoral nerve palsy (4). However, the average incidence of sciatic nerve injury following hip dislocations is 10 % in the medical literature. Partial recovery of sciatic nerve in these injuries is higher than 60 %, although it may not recover completely in delayed reduction (15). It seems that the elapsed time between injury and reduction affects the outcome of nerve palsy. According to this hypothesis a study was conducted by Hillyard et al. that showed transferring of patients with a dislocated hip between hospitals (like our reported patient) have a greater risk of sciatic nerve injury. In this study, the time to reduction was significantly longer in patients with major motor injuries (16). There is discrepancy in the literature regarding to the indications for surgical exploration of nerve injury after hip dislocation; however, most of them recommend that there is no need for surgical treatment unless the patient with normal nerve function experiences deterioration of its function after closed reduction (17). In the presence of nerve palsy, early rehabilitation and follow-up with periodic electromyography studies at one and five months after trauma are recommended (18). It`s important to examine neurologic function of the limb before reduction of a dislocation, although examination at the time of injury is difficult. This case emphasizes that early recognition of anterior hip dislocation and prompt closed reduction can relieve distortion of the nerve from a dislocated femoral head and prevent femoral nerve palsy.
